# Designing theoretically-informed implementation interventions

**DOI:** 10.1186/1748-5908-1-4

**Published:** 2006-02-23

**Authors:** The Improved Clinical Effectiveness through Behavioural Research Group (ICEBeRG)

**Affiliations:** 1 Centre for Health Services Research, 21 Claremont Place, Newcastle upon Tyne, NE2 4AA, UK

## Abstract

Clinical and health services research is continually producing new findings that may contribute to effective and efficient patient care. However, the transfer of research findings into practice is unpredictable and can be a slow and haphazard process. Ideally, the choice of implementation strategies would be based upon evidence from randomised controlled trials or systematic reviews of a given implementation strategy. Unfortunately, reviews of implementation strategies consistently report effectiveness some, but not all of the time; possible causes of this variation are seldom reported or measured by the investigators in the original studies. Thus, any attempts to extrapolate from study settings to the real world are hampered by a lack of understanding of the effects of key elements of individuals, interventions, and the settings in which they were trialled. The explicit use of theory offers a way of addressing these issues and has a number of advantages, such as providing: a generalisable framework within which to represent the dimensions that implementation studies address, a process by which to inform the development and delivery of interventions, a guide when evaluating, and a way to allow for an exploration of potential causal mechanisms. However, the use of theory in designing implementation interventions is methodologically challenging for a number of reasons, including choosing between theories and faithfully translating theoretical constructs into interventions. The explicit use of theory offers potential advantages in terms of facilitating a better understanding of the generalisability and replicability of implementation interventions. However, this is a relatively unexplored methodological area.

## Introduction

Clinical and health services research is continually producing new findings that may contribute to effective and efficient patient care. However, despite the considerable resources devoted to this area, a consistent finding is that the transfer of research findings into practice is unpredictable and can be a slow and haphazard process. Implementation research is the scientific study of methods to promote the systematic uptake of research findings into routine clinical practice, and hence to reduce inappropriate care. It includes the study of influences on healthcare professionals' behaviour, and methods to enable them to use research findings more effectively.

Ideally, the choice of implementation strategies would be based upon evidence from randomised controlled trials [1]. Healthcare practitioners and managers should be able to read a systematic review of several trials of an implementation intervention and reliably replicate some – or all – of the interventions in their own settings, and be confident of what will happen as a consequence. However, this is not currently the case. This is partially due to the manner in which trials are typically reported, as well as the lack of contextual detail included in reports of systematic reviews.

Systematic reviews of implementation trials conducted to date have categorised interventions on an empirical basis with reviews of interventions such as audit and feedback [2], reminders [3], and outreach visiting [4]. Other reviews have examined the range of interventions used to deliver a common message format, such as clinical practice guidelines [5]. All of these reviews produce a consistent message – all interventions, both within and across categories, are effective some, but not all of the time, producing a range of effect sizes from no effect through to a large effect. Unfortunately, another consistent finding from these reviews is that the possible causes of this variation are seldom reported or measured by the investigators in the original studies. Added to this is the fact that empirical interventions may be described using the same label in different studies (e.g., outreach visiting), but may not contain the same elements or be delivered in the same sort of manner. Thus any attempts to extrapolate from study settings to the real world are hampered by a lack of understanding of the key elements of individuals, interventions, and the settings in which they were trialled. An analogy from clinical medicine is described in Box 1.

One way of addressing a situation such as this is to tackle the issue empirically by examining all relevant combinations of the perceived important and modifiable elements of interventions to determine which contribute to a successful intervention.

Using audit and feedback as an example (Table 2), then varying only five elements produces 288 combinations, and this is before any replication of studies or the addition of other potential elements of an intervention, such as educational meetings or outreach visits. Given the multiplicity of factors that would need to be addressed, such an approach is not feasible.

**Table 1:** The Red Pills

Imagine an initial trial of a drug to reduce the likelihood of acute stroke in high-risk patients, where the drug is described as "the red pill" rather than in terms of its pharmacological properties. Over two to three years the "red pill" produces positive outcomes across a range of randomised controlled trials of patients at high risk of stroke. It is trialled in patients with moderate risk and low risk, again producing positive outcomes. Clinicians are impressed by the "red pill's" (unknown) properties and so begin to investigate its role in the treatment of a range of other conditions, though these are chosen on an ad hoc basis as there is no underlying rationale for its use. Equally impressed by the effects of red pills, a number of pharmaceutical companies launch other versions of red pills – the magenta pill, the crimson pill, and the vermillion pill. After ten years of trials the Cochrane Collaboration Red Pill Review Group begins to conduct systematic reviews of the effectiveness of "red pills" in the treatment of patients with stroke, asthma, epilepsy, and migraine to establish the generalisable messages about the effectiveness of "red pills."

**Table 2:** Modifiable elements of audit and feedback

1. Content: Comparative or not, anonymous or not?
2. Intensity: Monthly, quarterly, semi-annually, annually?
3. Method of delivery: By post, peer, or non-peer?
4. Duration: Six months, one year, or two years?
5. Context: Primary care or secondary care?

Another way to address this situation would be to identify studies using audit and feedback, for example, which were successful in achieving desired outcomes, and comparing them with unsuccessful studies using the same implementation approach. Synthesising successes and failures in this manner could provide valuable insights as to which study features/components distinguish them. However, given the reporting limitations of systematic reviews and their component trials described above, there may not be data of sufficient breadth and detail to be able to make meaningful comparisons [6].

An alternative is to use a theoretical approach to conceptualise the important factors and their inter-relations. Clinical practice can be described in terms of general theories relating to human behaviour [7]. However, theory has not been commonly used in the field of implementation research. Within a review of 235 implementation studies only 53 used theory in any way – to inform study design, develop or design the implementation intervention, and/or describe or measure elements of process for post-hoc interpretation – and only 14 were explicitly theory-based [8]. For this subset of studies it was difficult to draw clear conclusions, as "the level of reporting of both the theories used and the design of interventions was generally quite poor." Although there are no empirical data to illuminate why theory has not been used more extensively, factors such as researchers' lack of awareness of behavioural theories, the difficulty in locating and choosing theories, the absence of rigorous testing of theories, and the lack of readily available measures could all be factors.

Studies of interventions to promote behaviour change in healthy people have explicitly used theoretically-based interventions [9,10]. For example, a meta-analysis of theoretically-based interventions to change sexual behaviour to reduce HIV risk found a reliable effect (on self-reported behaviour), unlike interventions based on intuitive clinical models [11]. A trial in the same area, but using clinical outcomes, also demonstrated a positive effect of a theoretically-based intervention [12].

A theoretical approach has been advocated by others [13,14] and offers the advantage of a generalisable framework within which to represent the dimensions that implementation studies address. In doing so, it informs the development and delivery of interventions, guides their evaluation, and allows exploration of potential causal mechanisms. Within this paper, we briefly define theory, illustrate how it can be used to develop change interventions for healthcare professionals, and discuss the pros and cons of using theory in implementation research. The overall argument is that better evaluations of what does and does not work in implementation research should be more likely with the explicit use of theoretically-informed interventions. Also, we recognize that a considerable amount of expertise in theory use by researchers exists in areas outside of the broader health and health care field. Thus, more work needs to be done to move the implementation research field forward, and this paper represents an effort to move this research agenda forward.

### What is a theory?

A *theory *is an organized, heuristic, coherent, and systematic articulation of a set of statements related to significant questions that are communicated in a meaningful whole [15] for the purpose of providing a generalisable form of understanding. It describes observations, summarizes current evidence, proposes explanations, and yields testable hypotheses. It represents aspects of reality that are discovered or invented for describing, explaining, predicting and controlling a phenomenon [15,16].

Theories can be described in terms of their scope. A *metatheory *is a theory about theory. A *grand or macro theory *is a very broad theory that encompasses a wide range of phenomena. It is a general construction about the nature and goals of a discipline. Grand theories are substantially non-specific and are made up of relatively abstract concepts that lack operational definitions, as well as relatively abstract propositions that are not amenable to direct empirical testing [17,18]. They tend to be developed through thoughtful and insightful appraisal of existing ideas or creative leaps beyond existing knowledge. Some scholars use the terms 'grand theory' and 'conceptual model' interchangeably because of their high level of abstraction [19]. *Mid-range theory *is more limited in scope, less abstract, addresses specific phenomena, and reflects practice. It encompasses a limited number of concepts and a limited aspect of the real world. Mid-range theories are made up of relatively concrete concepts that are operationally defined and relatively concrete propositions that can be empirically tested. Mid-range theory is designed to guide empirical inquiry. A *micro, practice or situation-specific theory *(sometimes referred to as prescriptive theory) has the narrowest range of interest and focuses on specific phenomena that reflect clinical practice, and are limited to specific populations or to a particular field of practice.

A theory can be explicit or implicit. Explicit theories are of the type described above. Implicit theories are personal constructions about particular phenomena, such as how to change health care practitioner behaviour, which resides in individuals' minds, and are assumed to be an aspect of meta-cognition – knowledge about one's own thinking. Operationalising an explicit theory can be compared to cooking, using the step-by-step instructions in a cookbook, whereas operationalising implicit theory is more akin to an experienced cook who knows the basic components, how they interact and how many pinches or handfuls of ingredients are required to produce the desired product. Successful intervention studies can result from experienced and knowledgeable researchers applying their implicit theories, assuming they are operationalised correctly, but are difficult for a naïve (or even an experienced) researcher to reproduce. Explicit theories have the advantage of transparency, reproducibility, testability, exploration of causal mechanisms, and generalisability. Although the use of theory requires its own set of skills, the use of explicit theory also allows use by researchers who have accumulated less implicit knowledge in the intervention "kitchen."

### Choosing theories

There is a bewildering range of theories from which to choose. Given this, an explicit process could be helpful in guiding one's choice. Theories analysis has been proposed as an explicit process to help guide the choice of theory. A series of considerations in a theory analysis [19] are shown in Table 3.

**Table 3:** Choosing theories

• **Determine the origins of the theory.**
The "origins of a theory" refers to the original development of the theory. Who developed it? Where are they from (institution, discipline)? What prompted the originator to develop it? Is there evidence to support or refute the development of the theory?
• **Examine the meaning of the theory.**
The meaning of a theory has to do with the theory's concepts and how they relate to each other. What are the concepts comprising the theory? How are the concepts defined? What is the relationship between concepts?
• **Analyze the logical consistency of the theory.**
The logical adequacy of a theory is the logical structure of the concepts and statements. Are there any logical fallacies in the structure of the theory?
• **Consider the degree of generalisability and parsimony of the theory.**
Generalisability refers to the extent to which generalizations can be made from the theory. Parsimony refers to how simply and briefly a theory can be stated and still be complete in its explanation of the phenomenon in question.
• **Determine the testability of the theory.**
Can the theory be supported with empirical data? A theory that cannot generate hypotheses that can be subjected to empirical testing through research is not testable.
• **Determine the usefulness of the theory.**
Usefulness of the theory is about how practical and helpful the theory is in providing a sense of understanding and/or predictable outcomes.

Appraising theories against these dimensions (Table 3) will still the leave the user with significant choice. It is also important to consider the theory that is most applicable given the clinican's behaviour and the stakeholders who are targeted for behaviour change. For example, focusing on an individual physician as the agent of change will lead to disappointing results if the capacity to change is solely within the control of the Chief of Staff at a hospital – or a regional health authority. This would have a significant impact on the type of theory one would choose to guide or frame an intervention (e.g., from a theory targeted at an individual to something like communication theory). Examples of candidate theories include the Theory of Planned Behaviour, Operant Conditioning, and Implementation Intentions. Other theories are discussed by authors such as Robertson, Walker and Grol et al. [20-22]

Having undertaken such analyses there is another set of considerations that can guide the decision on which theory to use to inform the development of healthcare professional behaviour change interventions. These are largely pragmatic in nature. Given that implementation researchers are probably not interested primarily in theory testing, use of a theory with validated constructs and well-established means of measuring the constructs would be both straightforward and parsimonious in terms of designing and operationalising an intervention trial. Also, it will be better to work with theories that have been evaluated rigorously, [22-24] ideally within a similar setting as the intervention trial under consideration.

### Using theory to develop implementation interventions

Having considered the role of theory and discussed some of the considerations in selecting a theory to work with, the next step is to consider how using theory can influence the development of implementation interventions.

It is possible that implementation interventions may be chosen merely because they represent either what has been done before or what is judged feasible. These interventions represent an "off-the-shelf" option that is not informed by any explicit theory or prior analysis of the situation, but is merely informed by, at most, researchers' implicit theories or intuitions. In this situation the results are likely to be uninformative beyond the single setting of application.

Beyond such "off-the-shelf" interventions there is a continuum of contextualization – the degree to which an intervention is matched to the circumstances of its application – to be considered. For example, interventions ranging from a considerable degree of contextualisation, where an intervention is relevant to a small number of settings – to much less contextualisation, where an intervention is relevant to a wide range of settings. The latter intervention, one that can be applied to diverse contexts, uses a more or less general, mid-range theory.

An example of a contextualised intervention, constructed by attention to the details of a single specific application, and using implicit theory, is shown in Table 4.

**Table 4:** An empirical approach to cholesterol-lowering therapies in patients with diabetes.

There is a concern that primary care physicians are under-prescribing cholesterol-lowering therapy to patients with diabetes.
Physicians are interviewed leading to the identification of specific barriers to this behaviour: a lack of knowledge of recent research evidence about cholesterol-lowering therapy and concerns about serious drug side-effects.
This leads to an intervention that has two components: an educational component summarising recent relevant research evidence about cholesterol-lowering therapy and the presentation of prevalence data of the drug side-effects and their consequences.

There is no expectation that the intervention in Table 4 will provide a framework for addressing the adoption of other desirable prescribing behaviours (e.g., barriers around patient compliance), or for addressing different behaviours in other clinical areas. Furthermore, in this situation there would be no rigorously tested methods for operationalising variables and no outcome measures on variables other than those the researcher judged important in his/her implicit theory. In a situation such as this, "theorizing" about the intervention is heavily bound to the context of the practical problem that motivated it, and there can be little or no attempt to build a more explicit and generalisable theory.

At the other end of the contextualisation continuum, interventions can be based on general theories that have been developed and tested outside a particular application of interest, although they may still have been inspired by particular practical problems. These are what we referred to earlier as grand or macro theories: they formally address generalized principles and aspire to cross contexts. They can be wholly de-contextualized, in that they may apply to a wide variety of situations that obey common causal principals but are functionally unrelated.

As an example of using a mid-range theory, our group has experience using the Theory of Planned Behaviour (TPB) [25] as a process evaluation tool around intervention trials. TPB proposes a causal mechanism, where intention is the precursor of behaviour and is influenced by individuals' attitudes to the behaviour, their subjective norms about the behaviour, and perceptions of control over the behaviour. This theory has been successfully applied in a wide range of health and educational settings [26,27]. Table 5 shows a re-working of the above example about prescribing lipid-lowering therapy to patients with diabetes, applying the TPB.

**Table 5:** A theory-based approach to cholesterol-lowering therapies in patients with diabetes.

There is a concern that primary care physicians are under-prescribing cholesterol lowering therapy to patients with diabetes.
After initial interviews physicians are surveyed with an instrument based upon the constructs of the theory of planned behaviour. The results indicate that their *intention *to prescribe is significantly related to their *attitudes *to the benefits of lipid-lowering therapy in patients with diabetes and to their perceptions of the views of their hospital specialist colleagues (*subjective norms*).
This leads to an intervention that has two components: a *persuasive message *delivered by a *respected secondary care specialist*.

It is generally the case that empirical support for mid-range (de-contextualized) theories arises primarily from outside the immediate context of their current application. A highly contextualized theory or an implicit theory is likely to be applicable to only one problem, while grand or mid-range theories will tend to produce greater information per investigation because the empirical data collected can be applied beyond the specific circumstances of testing. In general, a theory can shift from being a micro theory to a mid-range or ultimately grand or macro theory, the more it is successfully applied to another, different specific problem. However, this increase is not linear because at some point, after multiple successful applications across a range of situations, another successful application does not prove any more about the theory (although it can continue to solve problems).

### Why theory may not work

There are three main reasons why an intervention-based on explicit theory may not work. First, a theory may be inadequate. Faulty research or logic may result in theories with inappropriate concepts, unclear definitions, or relationships that do not withstand rigorous testing. Any intervention based upon such a theory is unlikely to be successful in a predictable manner.

Second, the choice of theory may not be appropriate to the specific context. For example, the Theory of Planned Behaviour is most appropriately applied in situations where the focus of interest is the planned behaviour of individual clinicians. If the problem is largely an administrative one, such as the functioning of an appointment system, then such a motivational theory may be of limited help in designing an intervention. If there is not an appropriate theory available, then it may be better to choose a practical/micro theory or an implicit theory rather than use a mid-range theory that does not fit the circumstances of the intervention.

Finally, the impact of an intervention based on theory can be influenced by how well it is operationalised (put into practice). Poorly operationalised theories can produce two problems. First, if an intervention has no effect it will not be clear whether this is due to a genuine lack of effect with the intervention delivered as planned, or whether it is the consequence of poor operationalisation. Secondly, by failing to identify important mediating variables, it can hurt practice because a theory that is poorly operationalised has the potential to divert attention away from the factors that are actually influencing outcomes in the particular context.

### The role of theory in other aspects of design and statistical analysis

The preceding section has focused on the role of theory in guiding the development of interventions. However, theories also have practical consequences for the choice of study outcomes and for the analysis of study outcomes in the evaluation of interventions.

#### Using theories to guide the choices of study outcome

The absence of an explicit theory about the mechanism of the intervention can lead to difficulties. Lack of theoretical guidance can lead to a restricted focus on, for instance, the single end point of mortality, or other clinical outcomes that researchers feel are incontrovertibly important; thus from a negative trial, nothing is learned that could improve the intervention or take the research forward. An illustration of this, based on the contextualised intervention in Table 4, is presented in Table 6.

**Table 6:** The problem of a lack of an explicit theoretical framework

The intervention (see Table 4) using an educational component summarising recent relevant research evidence about cholesterol-lowering therapy and the presentation of prevalence data of the drug side-effects and their consequences, is found to have no effect on primary care physicians' prescribing behaviour.
However, measurement of the proposed mediating variables (knowledge of recent research evidence about cholesterol-lowering therapy and concerns about serious drug side-effects) indicates that the educational intervention did change both knowledge and physicians' concerns about side-effects. Therefore, at one level the intervention was successful, but it is now known that changing these two variables is not sufficient in itself to change the behaviour. This focuses the next phase of the research on other barriers that may not have been identified by the earlier interview study.
Conversely, in a parallel study, the educational intervention did not alter knowledge and concerns. Therefore, the possibility still holds that changing these variables will change behaviour, but it is clear that the educational strategy was insufficient to alter knowledge and opinions.

By contrast (and especially in observational studies) the absence of an explicit theory about the mechanism of the intervention can lead to the measurement of a large number of variables because researchers have little guidance about the likely consequences of intervening.

In the former instance, there is a risk of underestimating the effects of the intervention, particularly for randomized experiments that are often under-powered to begin with. In the latter instance, researchers encounter the worst problems of poorly specified models for correlated outcomes, over-fitting to samples, and poor control of Type I error-rates. Strong theories provide a clear framework for deciding what to measure.

### Fishing and the error rate problem

There have been decades of debate about the best way to handle the problem of multiple comparisons. Authors often report many statistical tests in a single paper, such as when pair-wise comparisons between two groups of participants are repeated for many measured outcomes, or a few outcomes are compared for several different groups. Conventional critical appraisal in such cases is that if the probability of a false positive conclusion is held at the usual Type I error rate of 5% for each of these tests, the probability that any one of them will be falsely declared significant is larger than 5%. One solution is to lower the risk of Type 1 error for each individual test (e.g., 1% for each of five tests), so that the study-wise risk is held at 5%, but such an approach will, for any given size, lower the power of the study.

By contrast, a theory offers protection against inflated study-wise error without threatening statistical power. Type I errors are problems of sampling error. That is, even if the null hypothesis is true, the random composition of a single sample can produce what appear to be positive effects. However, sampling error produces false-positive findings in either direction for any pair of variables, more or less randomly. Whilst replication of studies represents a sound protection against Type I errors, theories are particularly helpful in guiding the order of importance in terms of outcomes and effects, and the expected direction and theory-based empirical work can tell you the likely strength of effects.

### The tyranny of bivariate effects

Many literatures are dominated by bivariate tests that assess isolated "main effects" of various predictors on outcomes. A perusal of the literature may show that "A, B and C are known to affect Y", but often A, B and C were tested in separate analyses, or in separate studies. If A, B and C are correlated, as they often are likely to be in implementation research, this is a problem for two reasons. Firstly, overlapping covariance with Y means some amount of the "separate" effects of A, B and C is really the same effect discovered three times. Secondly, a test of all three variables together would not replicate the effects that were observed separately. How they differ depends how they are arranged in the model.

The simultaneous measurement and testing of correlated predictors does produce a new kind of uncertainty because now the answer depends on model specification. However, in the presence of a strong theory to guide the choice of relevant variables and their relationships, such studies produce more knowledge than would be obtained by the same number of subjects involved in separate tests of each predictor because it clarifies relationships between predictors and also possible interaction effects.

## Conclusion

Systematic reviews of implementation research point to limitations in the conceptualization, design and reporting of implementation trials that limit their generalisability. The aim of a randomised controlled trials (RCT) methodology is to evaluate the effectiveness of interventions to change the behaviour of health care professionals and, thereby, improve health outcomes. In this paper, we have argued that RCTs of interventions that aim to change behaviour can be more effective if they are based on explicit 'mid-range' theories that specify measurable mediators of behaviour change. Use of such theories can potentially lead to the more effective development of interventions by generating knowledge that is generalisable to a range of clinical contexts and behaviours, by generating data that can be analysed more efficiently and effectively, and that will provide a better understanding of how and why an intervention succeeded or failed.

Because explicit theories are available in the published research literature as formal statements containing definitions of constructs and their proposed interrelationships, the conceptual basis of theories is accessible for use by the research community. This provides a transparent basis for the development and evaluation of interventions and is, thus, preferable to the use of implicit theories.

Theory can be used to achieve the accumulation of generalizable knowledge about the processes underlying successful or unsuccessful interventions. However, this approach is fairly new in the area of health care professional behaviour change, an area that has, to date, been largely a-theoretical or, based on implicit theories. Given this novelty it is likely that there will be problems pursuing a theoretical path. It is reasonable to assume that theories applied outside of healthcare may be successfully applied within. However, there are two reasons why theories may not perform in precisely the same manner when applied to healthcare settings: the agency relationship and the fact that the consequences of a clinician's behaviour are often experienced not by them but by their patients. The agency relationship in health care refers to the observed asymmetry in terms of training, knowledge and experience along with patients' vulnerability, due to illness, that accounts for the considerable influence, desirable or otherwise, that clinicians have on patients' treatment decisions. Both of these considerations could alter the strength of relationships between theoretical constructs.

It is also possible that the health services research challenges of using theories may impose limits on whether and how quickly the area can move forward. For instance, the challenges of data collection within the complex situation of health care delivery are daunting. Therefore, to move forward it is necessary to build up a body of knowledge in this field, together with empirical evidence to support the use of theory-informed interventions and theory-informed evaluations. A starting point is to work with a small number of theories and to build up expertise in how best to apply them in this field. This approach offers the potential to streamline the processes of intervention development. However, it represents a substantial change in thinking about implementation trials in ways that are only just beginning to be articulated, and necessitates a long-term research effort to answer both the theoretical, as well as the practical research questions.

Because mid-range theories as we have described them include specifications for operationalising the relevant constructs, the capacity to measure theoretical constructs is within the reach of any researcher who thoughtfully reads the relevant literature. Collaborating with researchers in other disciplines, who have relevant expertise and experience, is an effective way of fast-tracking through this process. There is already considerable experience in applying theory amongst researchers in other disciplines, relating to contexts other than health care. The applicability of theories across these contexts makes a vast amount of existing expertise available to the clinical community that could contribute to moving this field forward in an interdisciplinary manner.

## Contributors

The members of the ICEBeRG Group are:

Doug Angus, School of Management, University of Ottawa

Melissa Brouwers, Dept Clinical Epidemiology and Biostatistics, McMaster University

Michelle Driedger, Dept of Geography, University of Ottawa

Martin Eccles, Centre for Health Services Research, University of Newcastle upon Tyne

Jill Francis, Health Services Research Unit, University of Aberdeen

Gaston Godin, Groupe de recherche sur les comportements de santé, Université Laval

Ian Graham, School of Nursing, University of Ottawa

Jeremy Grimshaw, Clinical Epidemiology Program, Ottawa Health Research Unit, Ottawa, Department of Medicine, University of Ottawa

Steven Hanna, CanChild Centre for Childhood Disability Research, McMaster University

Margaret B Harrison, School of Nursing, Queens University

France Légaré, Unité de recherche évaluative, Centre Hospitalier Universitaire de Québec

Louise Lemyre, Institute of Population Health, University of Ottawa

Jo Logan, School of Nursing, University of Ottawa

Rosemary Martino, Faculty of Medicine, University of Toronto

Marie-Pascale Pomey, School of Management, University of Ottawa

Jacqueline Tetroe, Ottawa Health Research Unit, Ottawa

## Competing interests

The author(s) declare that they have no competing interests.

### Authors' contributions

The idea for this paper came from a meeting involving all of the members of the group. Martin Eccles, Steve Hanna, Jo Logan, Ian Graham and Jacqueline Tetroe wrote the drafts. All members of the group discussed and offered comments on the contents of the drafts and approved the final manuscript.
